# Sulforaphane Preconditioning Sensitizes Human Colon Cancer Cells towards the Bioreductive Anticancer Prodrug PR-104A

**DOI:** 10.1371/journal.pone.0150219

**Published:** 2016-03-07

**Authors:** Melanie M. Erzinger, Cédric Bovet, Katrin M. Hecht, Sabine Senger, Pascale Winiker, Nadine Sobotzki, Simona Cristea, Niko Beerenwinkel, Jerry W. Shay, Giancarlo Marra, Bernd Wollscheid, Shana J. Sturla

**Affiliations:** 1 Department of Health Sciences and Technology, ETH Zurich, Zurich, Switzerland; 2 Department of Biology, Institute of Molecular Systems Biology, ETH Zurich, Zurich, Switzerland; 3 Department of Biosystems Science and Engineering, ETH Zurich, Basel, Switzerland; 4 SIB Swiss Institute of Bioinformatics, Basel, Switzerland; 5 Department of Cell Biology, University of Texas Southwestern Medical Center, Dallas, Texas, United States of America; 6 Institute of Molecular Cancer Research, University of Zurich, Zurich, Switzerland; 7 BioMedical Proteomics Platform (BMPP), ETH Zurich, Zurich, Switzerland; Colorado State University, UNITED STATES

## Abstract

The chemoprotective properties of sulforaphane (SF), derived from cruciferous vegetables, are widely acknowledged to arise from its potent induction of xenobiotic-metabolizing and antioxidant enzymes. However, much less is known about the impact of SF on the efficacy of cancer therapy through the modulation of drug-metabolizing enzymes. To identify proteins modulated by a low concentration of SF, we treated HT29 colon cancer cells with 2.5 μM SF. Protein abundance changes were detected by stable isotope labeling of amino acids in cell culture. Among 18 proteins found to be significantly up-regulated, aldo-keto reductase 1C3 (AKR1C3), bioactivating the DNA cross-linking prodrug PR-104A, was further characterized. Preconditioning HT29 cells with SF reduced the EC_50_ of PR-104A 3.6-fold. The increase in PR-104A cytotoxicity was linked to AKR1C3 abundance and activity, both induced by SF in a dose-dependent manner. This effect was reproducible in a second colon cancer cell line, SW620, but not in other colon cancer cell lines where AKR1C3 abundance and activity were absent or barely detectable and could not be induced by SF. Interestingly, SF had no significant influence on PR-104A cytotoxicity in non-cancerous, immortalized human colonic epithelial cell lines expressing either low or high levels of AKR1C3. In conclusion, the enhanced response of PR-104A after preconditioning with SF was apparent only in cancer cells provided that AKR1C3 is expressed, while its expression in non-cancerous cells did not elicit such a response. Therefore, a subset of cancers may be susceptible to combined food-derived component and prodrug treatments with no harm to normal tissues.

## Introduction

Cancer drugs are often associated with severe side effects that limit dosing potential, therefore prodrugs that require bioactivation in target cells are actively pursued as a strategy to promote therapeutic selectivity [1]. To further differentiate between target and non-target cells, particularly for enzyme-activated prodrugs, a novel alternative approach is to selectively precondition cancer cells with non-toxic amounts of a natural bioactive compound to safely enhance drug susceptibility [2]. These compounds often up-regulate drug metabolizing enzymes that bioactivate drugs, therefore despite low exposures, they may significantly impact therapy outcomes [3]. Unlike drug-drug interactions, food-modulated changes in drug metabolism that influence drug efficacy in cancer therapy have rarely been addressed.

Isothiocyanates such as sulforaphane (SF) are derived from cruciferous vegetables, are bioavailable in the colon [4], and modulate gene expression of a large number of xenobiotic-metabolizing and antioxidant enzymes [4–6]. To a large extent, this process is mediated by the transcription factor nuclear factor erythroid 2-related factor 2 (Nrf2) [7]. The influence of SF on gene transcription and protein expression has been characterized in rodent models and human cell lines from different tissue origin [8–18], including four studies entailing proteomic approaches [14, 16–18]. SF reacts with cysteine residues of the Nrf2 repressor Keap1, resulting in nuclear translocation of Nrf2 and binding of the transcription factor to DNA [7]. Gene expression is affected mainly for genes that code for phase II and detoxification enzymes, but also cellular NADPH-regenerating enzymes, antioxidants, or xenobiotic-metabolizing enzymes [14–18]. Most translational applications of SF aim to exploit the regulation potential for deactivating electrophiles and reactive oxygen species in healthy or pre-malignant cells for cancer prevention [15, 19].

While SF or high levels of Nrf2 may contribute to chemoresistance [7, 20], the opposite relationship has also been observed, with key differences being mechanism of drug action and cell characteristics [21]. Most known instances involve a direct therapeutic function of SF in a drug-like manner, however, there is limited knowledge regarding influences of non-toxic, low concentrations of SF potentially achieved by the diet. A process of particular relevance is how transcriptional activation of drug-activating enzymes may promote the action of cancer prodrugs. In this regard, it has been observed that when cancer cells (breast TD47D) were treated with SF, NAD(P)H:quinone oxidoreductase 1 (NQO1), an activator of mitomycin C (MMC), was induced and cells were sensitized to MMC [22]. In a follow-up study, dimethyl fumarate was used as NQO1 inducer, and the initial SF findings were confirmed *in vivo*. Importantly, there were no observed increases in adverse toxicities, motivating further study of how diet-relevant enzyme induction may impact prodrug activity [23]. These data, together with the established bioavailability of SF in the human colon, suggested to us the relevance of characterizing proteome-wide changes in human colon cancer cells exposed to non-toxic doses of SF using stable isotope labeling with amino acids in cell culture (SILAC) [24], a metabolic labeling approach that allows the quantification of thousands of proteins with high precision and sensitivity [25].

**Fig 1:**
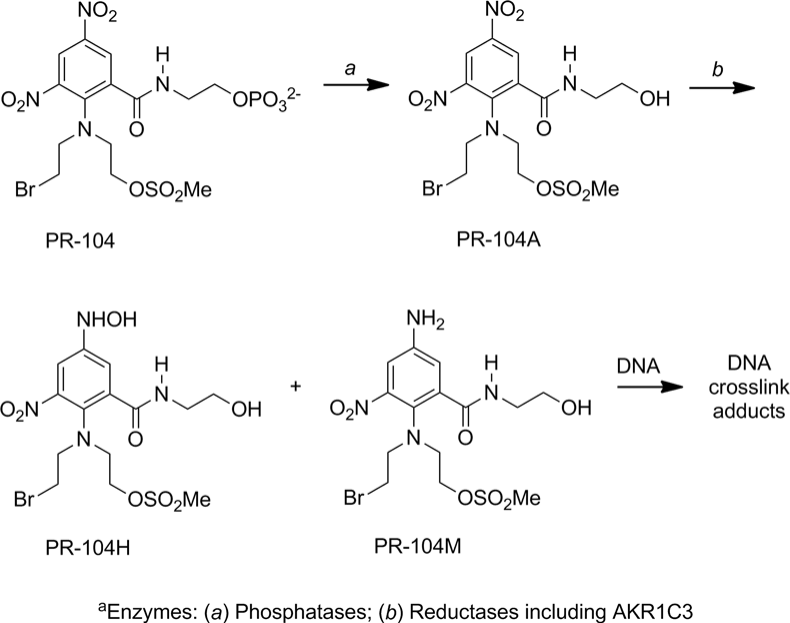
Activation of the bioreductive prodrug PR-104A, a metabolite of the dinitrobenzamide mustard pre-prodrug PR-104, by aromatic nitro-reduction. *In vivo*, PR-104 is hydrolyzed to PR-104A, which is further reduced to metabolites that form cytotoxic ICLs.

PR-104A is the prodrug metabolite of the dinitrobenzamide mustard pre-prodrug PR-104 (Fig 1) [26–29]. In previous studies, the enzyme aldo-keto reductase (AKR) 1C3 has been established to mediate the aerobic activation of PR-104A [29, 30]. This enzyme is a member of the AKR enzyme superfamily, comprised of ketosteroid reductase enzymes that regulate the production of androgens, estrogens, and progestins [31] and its expression is regulated by the Nrf2/Keap1-pathway [29]. PR-104 has been involved in a number of early clinical trials for cancer therapy [32–35], however, a potential beneficial effect of chemically induced enzyme expression on PR-104A cytotoxicity has not been reported.

To determine the influence of a low SF concentration that might be exploited for the modulation of drug efficacy, we analyzed proteome-wide changes upon SF preconditioning in HT29 colon cancer cells by SILAC [24]. On the basis of these data, we formulated the hypothesis that PR104A activity may be promoted in cancer cells. Therefore, we tested the impact of SF preconditioning on PR-104A cytotoxicity in these and several other established colon cancer cell lines and compared to immortalized but normal diploid human colonic epithelial cells (HCEC) as a model for healthy colon tissue. A range of data from cellular uptake, enzyme abundance, activity in different cell types and knock-down assays suggest a model involving SF-promoted sensitization on the basis of increased AKR1C3 protein abundance and activity, and cellular characteristics that favor the positive interaction.

## Materials and Methods

### Materials

Cell culture medium and supplements were from Invitrogen (Life Technologies) and chemicals from Sigma Aldrich, if not otherwise specified. Stock solutions of R-sulforaphane (LKT Laboratories) and PR-104A (Proacta) were prepared in DMSO, chlorambucil in ethanol. NADPH was from Calbiochem. Coumberone was generously provided by Prof. Dalibor Sames (Columbia University, NY, USA) and SN34037 by Prof. Bill Wilson and Dr. Adrian Blaser (Auckland Cancer Society Research Centre, University of Auckland, New Zealand). ON-TARGETplus Human AKR1C3 siRNA (SMARTpool) and ON-TARGETplus non-targeting pool siRNA were obtained from Dharmacon (Thermo Scientific). Lipofectamine RNAiMAX transfection reagent (Life Technologies) was used for siRNA transfection according to the manufacturer's protocol.

### Cells and Culture Conditions

HT29 cells were obtained from the Leibnitz-Institut DSMZ GmbH in January 2012. SW620, SW480, HCT116 and GP2d cells, described in [36, 37], were obtained from the Institute of Molecular Cancer Research (University of Zurich, Switzerland) in October 2013 and authenticated by the company Microsynth (Balgach, Switzerland) using short tandem repeat profiling in January 2015. HT29, SW620 and GP2d cells were grown in DMEM, SW480 cells were cultured in RPMI-1640 medium and HCT116 cells were grown in McCoy’s medium. All media were supplemented with 10% (v/v) fetal bovine serum and 100 units/mL penicillin and 100 μg/mL streptomycin. HCEC clones were obtained in August 2011 (HCEC1CT) and August 2013 (HCEC2CT) and grown under previously reported conditions, but under normoxia [38]. All cell lines have regularly been confirmed to be mycoplasma free. For SILAC experiments, HT29 cells were grown in lysine and arginine free DMEM (Silantes) that was either supplemented with 0.219 mM lysine (Lys0) and 1.14 mM arginine (Arg0) for the light medium (L), while the heavy medium (H) was supplemented with 0.219 mM [^13^C_6_^15^N_2_]-lysine (Lys8) and 1.14 mM [^13^C_6_^15^N_4_]-arginine (Arg10). For a complete incorporation of the isotope-labelled amino acids, cells were grown for a total of 24 cell population doublings (six passages, 4 cell population doublings each) in SILAC medium prior to the experiment. 24 h after seeding, cells grown in SILAC light medium were treated with 0.1% (v/v) DMSO and cells grown in SILAC heavy medium were treated with 2.5 μM SF for 48 h. All cell samples were prepared in triplicates.

### Protein Extraction

Cells were trypsinized, washed with PBS, and incubated at 4°C for 15 min with lysis buffer (150 mM NaCl, 50 mM Tris HCl, 1 mM EDTA, pH 7.4) containing protease inhibitors (Complete Mini, Roche Diagnostics). Lysate was sonicated and centrifuged, protein concentration determined by BCA assay (Thermo Fisher Scientific), and samples stored at -80°C.

### SILAC

Protein digestion, mass spectrometric analysis of SILAC samples and bioinformatics analysis was performed by adaptation of standard approaches (full details regarding method established for this study in S1 Appendix).

### AKR1C3 Activity

AKR1C substrate coumberone was used as activity probe together with a specific AKR1C3 inhibitor (SN34037) by modification of the method described in [30]. In 96-well plates for each sample, 40 μg of total protein were added to assay buffer (100 mM KPO4 buffer [pH 7] containing 250 μM NADPH) with or without 1 μM SN34037, and incubated 60 min, 37°C. The reaction was initiated with coumberone (30 μM final; DMSO 5% [v/v]). Fluorescence emission, due to the formation of coumberol, at 510 nm (385 nm excitation) was recorded on an infinite M200 PRO plate reader (Tecan) at 37°C. AKR1C3 activity was calculated as ΔRFU/min over 60 min (full coumberone metabolism in S1 Fig). Measurements performed in duplicate with biological triplicates; statistical analysis (mean values, student’s t-test) performed using GraphPad Prism 6.

### Western Blot Analysis

Cell lysates were separated on NuPAGE® 4–12% Bis-Tris gel at 200 V for 45 min in 1X NuPAGE® MES SDS running buffer (Life Technologies) and electrophoretically transferred to Amersham Hybond-P PVDF membrane (GE Healthcare) at 30 V for 60 min in 1X NuPAGE® transfer buffer (Life Technologies). PVDF membranes were blocked with 5%-milk-TBST at RT. The membranes were incubated with rabbit polyclonal anti-AKR1C3 antibody (1:2’000, Thermo Scientific), followed by incubation with anti-rabbit IgG HRP antibody (1:5’000, GE Healthcare). The protein was detected using a Pierce® ECL Western Blotting Substrate (Thermo Scientific). Treated membranes were exposed to X-ray film. The membrane was stripped, incubated with anti-actin antibody (1:1'000, Sigma Aldrich) and analyzed again. Protein bands were quantified with ImageJ software [39], and were adjusted for corresponding actin loading control.

### Drug Cytotoxicity

Cells were seeded in 96-well plates (HT29, HCEC, SW480: 1’500 cells/well; SW620: 1'000 cells/well; HCT116: 500 cells/well; GP2d: 5'000 cells/well). 24 h later cells were exposed to 2.5 μM SF (exception HCT116: 1 μM) corresponding to an IC_10_ or 0.1% (v/v) DMSO. After 48 h, medium was removed and increasing concentrations of drug in fresh medium were added. 24 h afterwards medium was replaced by fresh medium. 72 h later cell viability was assayed using the CellTiter-Glo® Luminescent Cell Viability Assay (Promega) according to the manufacturer’s protocol. The experiment was performed in triplicate. All luminescence values were normalized relative to vehicle-treated cells. The dose-response curves were fitted by a nonlinear regression function in GraphPad Prism 6. Statistical analysis was performed by extra sum-of-squares F test to test if dose-response curves in control and SF-pretreated cells statistically differ from each other.

### PR-104A Cytotoxicity with siAKR1C3

HT29 cells were seeded in 6-well plates (90’000 cells/well) and allowed to attach. 24 h later they were exposed to siAKR1C3 (120 pmol) and 2.5 μM SF or 0.1% DMSO simultaneously. After 48 h, medium was removed and three concentrations of PR-104A (0, 25, 100 μM) were added in fresh medium. 4 h after adding the drug, the medium was removed and a clonogenic survival assay was performed according to standard protocols [40]. After two weeks incubating, colonies were stained with Giemsa stain and counted. As a control, non-targeting siRNA (120 pmol) was used. The experiment was performed in triplicate. Statistical analyses (unpaired t-test with Welch’s correction) were performed using GraphPad Prism 6.

### SF Uptake

Intracellular SF concentrations in HT29 and HCEC1CT cells were measured by the HPLC-coupled cyclocondensation assay (reaction with 1,2-benzenedithiol), as previously described (further details in S1 Appendix) [41].

## Results

### Influence of SF on gene expression in HT29 colon cancer cells

**Fig 2:**
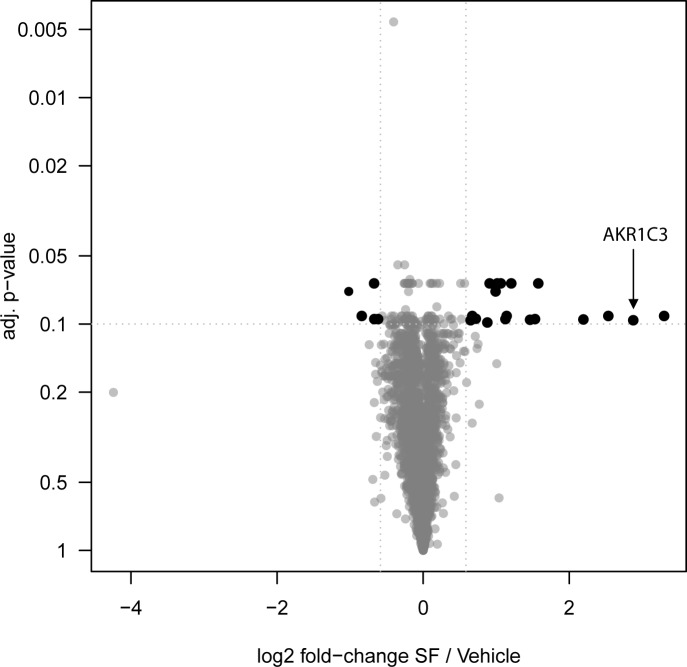
Volcano plot showing significant changes in the proteome following exposure of HT29 cells to 2.5 μM SF for 48 h, determined by SILAC. 2653 proteins (black and gray) were quantified and tested for significance. Among the 23 significantly regulated proteins (black), 18 were up-regulated and 5 down-regulated in abundance.

SILAC was used to investigate changes in abundance levels of cellular proteins upon treatment with a low concentration of SF in HT29 colon cancer cells. After treatment with 2.5 μM SF for 48 h, corresponding to an IC_10_ in HT29 cells (S2 Fig); we identified 23 differentially abundant proteins (Fig 2; Table 1). A preponderance of proteins involved in cellular metabolic and redox processes were found to be enriched, and enzymes characterized previously to mediate the activation of reductive prodrugs, such as AKR1C3, PTGR1, and NQO1 were amongst this group [22, 29, 42–44]. The largest fold change was evident for Cyclin-D1-binding protein 1, a negative regulator of transcriptional activation involving the E2F transcription factors [45], whereas several metabolic and redox-regulating proteins regulated by the transcription factor Nrf2, including AKRs and an aldehyde dehydrogenase, increased by 4.6-fold or higher. The large change in abundance for AKR1C3 (7-fold), previously implicated in the activation of PR-104A, invoked the possibility for drug sensitization. Therefore, we focused our further efforts in elucidating any SF-PR104A interactions and contributions of AKR1C3 as a molecular basis. A full list of all quantified proteins in the SILAC experiment, including their normalized H/L ratio, is provided in S1 Table.

**Table 1:** Fold changes in protein abundance for SF-treated HT29 cells compared to untreated counterparts.^a^

Protein Name	Gene Name	Fold Change Protein Abundance (SF/Vehicle)^b^
Cyclin-D1-binding protein 1	*CCDB1*	9.8
Aldo-keto reductase family 1 member C3^c^	*AKR1C3*	7.3
Aldo-keto reductase family 1 member B10^c^	*AKR1B10*	5.8
Aldehyde dehydrogenase, dimeric NADP-preferring	*ALDH3A1*	4.6
Glutamate-cysteine ligase catalytic subunit	*GCLC*	3.0
Sulfiredoxin-1 (EC 1.8.98.2)	*SRXN1*	2.9
Glutamate-cysteine ligase regulatory subunit	*GCLM*	2.8
Glucose-6-phosphate 1-dehydrogenase	*G6PD*	2.3
Prostaglandin reductase 1	*PTGR1*	2.2
UDP-glucose 6-dehydrogenase	*UGDH*	2.2
NAD(P)H dehydrogenase (quinone) 1	*NQO1*	2.0
Transketolase^c^	*TKT*	2.0
Thioredoxin reductase 1	*TXNRD1*	2.0
Fatty aldehyde dehydrogenase	*ALDH3A2*	1.9
Phosphoserine aminotransferase	*PSAT1*	1.8
Thioredoxin	*TXN*	1.6
6-phosphogluconate dehydrogenase	*PGD*	1.6
Glutathione Reductase, mitochondrial	*GSR*	1.6
Villin-1	*VIL1*	0.7
Beta-2-microglobulin	*B2M*	0.6
Creatine kinase B-type	*CKB*	0.6
Selenoprotein O	*SELO*	0.6
Tubulin polymerization-promoting protein family member 3 (TPPP/p20)	*TPPP3*	0.5

a. Incubating HT29 cells for 48 h with 2.5 μM SF twenty-three proteins were found to be significantly modulated (n = 3; p-value < 0.1, fold change < 0.67 or > 1.5).

b. Ratio H/L normalized

c. Proteins reported as non-differentiable protein groups.

**Fig 3:**
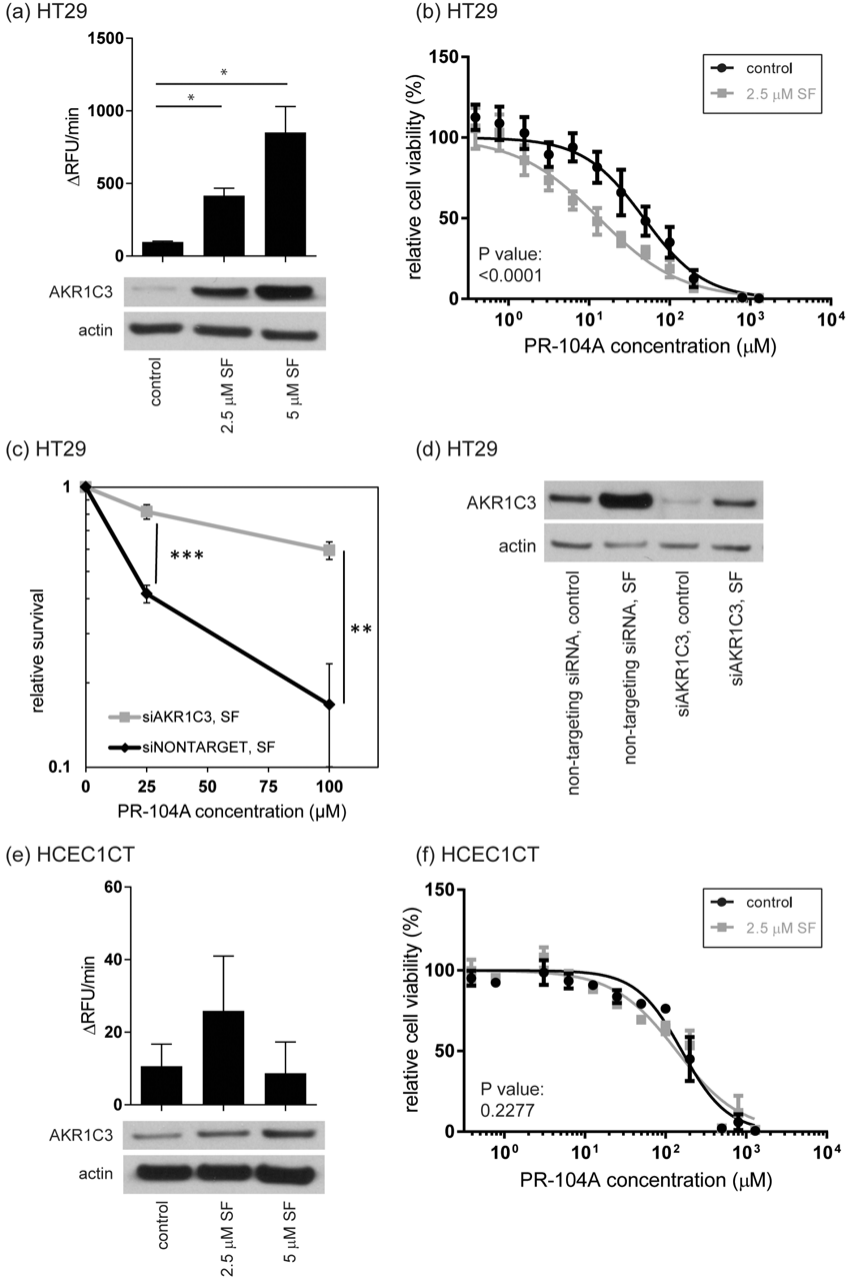
Influence of SF on AKR1C3 activity, protein expression, and PR-104A cytotoxicity. AKR1C3 activity and protein levels are shown in (a) for HT29 cells and in (e) for HCEC1CT cells treated 48 h with SF or DMSO (control). Bars represent mean values and error bars are standard errors. Statistical analysis was performed by an unpaired t-test with Welch’s correction. Experiments were performed in triplicate. (*: *p* < 0.05; RFU = relative fluorescence units). Modulation of cell viability is shown for HT29 in (b) and for HCEC1CT in (f) after cells were incubated 48 h with 2.5 μM SF or 0.1% DMSO (control) and incubated with increasing concentrations of PR-104A. Data shown are mean values ± SD from four (HT29) or three (HCEC1CT) biological replicates. An extra sum-of-squares F test was performed to test whether dose-response curves statistically differ from each other. The resulting p-values show that SF significantly impacts drug response in HT29 cells, and has no significant effect in HCEC1CT cells. (c) Clonogenic survival assay showing increased survival of HT29 treated with siRNA against AKR1C3 compared to cells treated with non-targeting siRNA after PR-104A treatment for 4 h. Cells were pretreated for 48 h with 2.5 μM SF together with siRNA. Each data point represents three independent experiments. Error bars show SD of the mean. Statistical analysis was performed by an unpaired t-test with Welch’s correction; **: *p* < 0.01, ***: *p* < 0.001. (d) Western Blot showing levels of AKR1C3 protein in HT29 cells treated with either non-targeting siRNA or siAKR1C3 with or without simultaneous SF treatment (control = 0.1% DMSO).

To independently confirm the increase in AKR1C3 abundance apparent in the SILAC study, Western Blot analysis was performed and it was found that AKR1C3 abundance was induced in HT29 cells in an SF dose-dependent manner (Fig 3A). To confirm higher AKR1C3 activity in HT29 lysates, we used a modified version of a method previously described for measuring specifically AKR1C3 activity in intact cells, involving coumberone as a substrate for all four members of the AKR1C family, and the specific AKR1C3 inhibitor SN34037 [30]. Similar to protein levels, AKR1C3 activity increased in a dose-dependent manner in HT29 cells (Fig 3A).

### Impact of SF-mediated AKR1C3 induction on drug toxicity in HT29 colon cancer cells

We found that by preconditioning HT29 cells with 2.5 μM SF for 48 h, followed by treatment with increasing doses of PR-104A, a significant 3.6-fold decrease in the EC_50_ of the drug was observed (Fig 3B; Table 2; *p* < 0.0001). Higher SF concentrations were tested for the preconditioning, but increasing toxicity of the pretreatment itself prevented the further use of these higher SF concentrations. As a control, this interaction was compared with the influence of SF preconditioning on the cytotoxicity of chlorambucil (CBL), an anticancer drug that also forms DNA interstrand cross-links (ICLs) but does not rely on enzymatic bioactivation. As expected, the cytotoxicity of CBL was unchanged (Table 2).

**Table 2:** PR-104A and CBL cytotoxicity in colon cell lines pretreated with SF.^a^

	HCEC1CT	HCEC2CT	HT29	SW620	SW480	HCT116	GP2d
PR-104A EC_50_ (μM) control^b^	160.1	82.5	48.0	23.3	129.4	104.2	84.8
95% confidence interval	128.8–198.9	66.3–102.8	34.4–67.1	18.0–30.1	109.1–153.4	81.8–133.0	44.2–162.6
PR-104A EC_50_ (μM) SF pretreated^b^	138.3	92.3	13.3	6.4	103.7	70.3	84.2
95% confidence interval	105.7–180.8	73.0–116.7	9.8–17.9	4.7–8.6	84.1–127.8	51.0–96.8	50.2–141.1
p-value^c^	0.2	0.8	<0.0001	<0.0001	0.3	0.2	1.0
CBL EC_50_ (μM) control^b^	77.7	30.7	49.3	36.9	41.3	26.2	23.2
95% confidence interval	56.7–106.5	22.7–41.6	32.6–74.7	20.8–65.7	33.4–51.1	21.2–32.5	14.4–37.5
CBL EC_50_ (μM) SF pretreated^b^	66.7	40.7	42.8	24.6	32.0	27.2	22.4
95% confidence interval	42.6–104.3	28.6–58.1	24.3–75.3	16.4–36.9	21.8–47.0	21.7–34.0	14.6–34.4
p-value^c^	0.7	0.5	0.9	0.5	0.1	0.9	1.0
Tissue origin	normal	normal	cancer	cancer	cancer	cancer	cancer
Basal mRNA AKR1C3^d^	nd^f^	nd^f^	11.3	11.3	4.2	5.0	9.1
AKR1C3 activity control (ΔRFU/min)^e^	10.6	116.4	96.6	217.1	‒23.6	5.9	28.0
AKR1C3 activity SF pretreated (ΔRFU/min)^e^	25.9	185.0	417.1	375.3	8.1	6.2	178.1

a. Cells were pretreated with either 0.1% DMSO (control) or 2.5 μM SF (exception: HCT116: 1 μM).

b. Corresponding 95% confidence intervals are shown below the EC_50_ values.

c. Extra sum-of-squares F test to test if dose-response curves in control and SF pretreated cell statistically differ from each other.

d. Basal mRNA level (log2) as assessed in [51], using Affymetrix U133 plus 2.0 arrays.

e. ΔRFU/min. Corresponding data shown in Fig 4A.

f. nd = not determined

To confirm if increased AKR1C3 abundance levels due to SF preconditioning of HT29 are responsible for the increased cytotoxicity of PR-104A, we performed RNA interference using a pool of four siRNAs against *AKR1C3*. A clonogenic assay was used to determine cytotoxicity of PR-104A in HT29 cells after simultaneous preconditioning with SF and siRNA against *AKR1C3* (Fig 3C and 3D, S2 Table). As control, non-targeting siRNA was used. Furthermore, varying incubation times and siRNA concentrations were tested to ensure that the SF-induced AKR1C3 expression was repressed by the siRNA against *AKR1C3* under the conditions described, and after 48 hours simultaneous preconditioning with SF and siRNA against *AKR1C3*, protein levels were similar to levels in untreated HT29 cells (data not shown). When the SF-induced AKR1C3 overexpression was down-regulated via RNA interference, the cytotoxicity of PR-104A was reduced, indicating that SF preconditioning lowered the PR-104A concentration required to achieve a similar cytotoxicity by inducing AKR1C3 abundance levels. These data support the previously established link between AKR1C3 abundance and PR-104A efficacy [29, 30], and furthermore confirms the same effect under conditions of chemical induction, i.e. with SF.

### Impact of SF preconditioning on drug toxicity in HCEC non-cancerous colonic cells

Promoting drug cytotoxicity in cancer tissue may be beneficial if the same interaction is absent or reduced in healthy counterparts. Therefore, the combination of SF and PR-104A was investigated in an immortalized human colonic epithelial cell line (HCEC) as a model for non-cancerous tissue [38]. HCECs (HCEC1CT and HCEC2CT, from two different individuals) are non-tumorigenic diploid cells that express epithelial as well as stem cell markers and were established from normal colonic mucosa. They do not carry mutations in hot spot genes such as *APC*, *KRAS*, or *TP53* [38]. AKR1C3 abundance levels measured by Western Blot were slightly induced upon SF treatment in HCEC1CT cells (Fig 3E), but to a lower extent than for the HT29 cells (Fig 3A). There was neither significant increase in AKR1C3 activity (Fig 3E) nor drug susceptibility (HCEC1CT: Fig 3F; Table 2; *p* = 0.2; HCEC2CT: Table 2; *p* = 0.8).

### SF uptake in HT29 and HCEC1CT cells

To examine whether the different response in HT29 vs. HCEC1CT cells might be related to cellular SF uptake, we analyzed SF uptake in HT29 and HCEC1CT by a cyclocondensation assay. This assay has the advantage of allowing quantification of free SF as well as SF bound to cellular thiols such as glutathione [41]. There were no significant differences in SF uptake (S3 Fig), suggesting the exclusion of increased uptake as a basis for the SF-PR104A interaction in HT29 but not HCEC cells.

### Impact of SF preconditioning on drug toxicity in additional colon cancer cell lines

To explore the generality and basis of SF preconditioning effects, cytotoxicity experiments were performed with additional colon cancer cell lines with different genetic backgrounds (Table 2). Similar to HT29, in SW620 the EC_50_ of PR-104A was significantly reduced 3.6-fold by SF preconditioning (*p* < 0.0001). As in HT29 cells, AKR1C3 abundance in SW620 cells was induced around 3-fold and activity increased 1.7-fold (Fig 4, S3 Table). In contrast, for HCT116, SW480, and GP2d cells, PR-104A cytotoxicity was not altered. This observation seems consistent with the fact that no AKR1C3 protein was detected in HCT116 and SW480 cell lysates, and nor did SF preconditioning induce protein abundance or activity (Fig 4, S3 Table). In GP2d cells, AKR1C3 was barely expressed; however, abundance and activity of this enzyme were not increased sufficiently by SF to alter PR-104A cytotoxicity (Fig 4, S3 Table). A clear trend was observed towards lower PR-104A EC_50_ values for cells with higher AKR1C3 activity (Fig 4C, R^2^ = 0.7063; *p* < 0.01). These results indicate that a combination of AKR1C3 basal levels and activity, as well as susceptibility to induction by SF, influence the propensity of cells to support the PR-104A-enhancing potential of SF. Therefore, genetic variation across primary healthy and tumor tissue, as well as different cancers, may be expected to contribute to individual responses.

**Fig 4:**
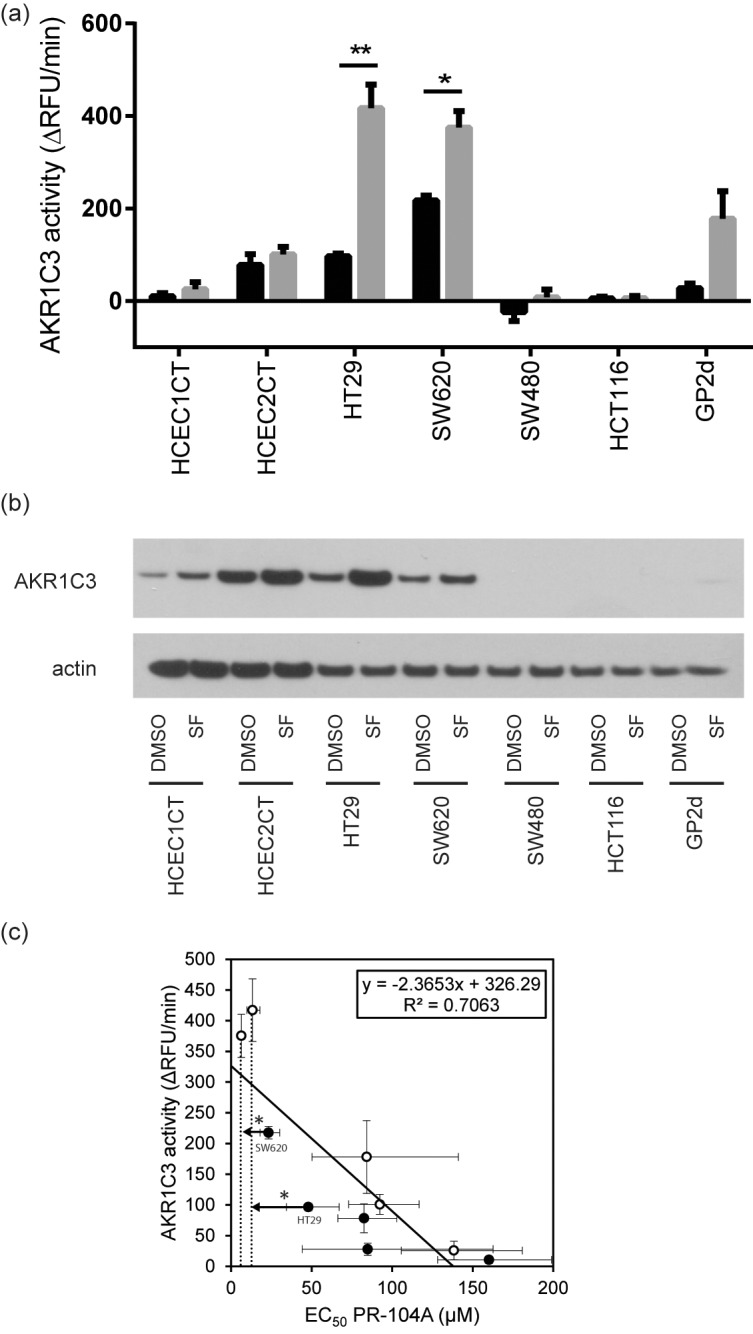
AKR1C3 enzyme activity and protein levels in different colon cell lines and correlation between AKR1C3 activity and PR-104A cytotoxicity. (a) AKR1C3 activity with (gray) and without (black) SF preconditioning for colon cell lines used in this study. Statistical analysis was performed by unpaired t-test with Welch’s correction; *: *p* < 0.05. (b) Western Blot for AKR1C3 protein with and without SF preconditioning (2.5 μM for 48 h). (c) Correlation between AKR1C3 activity and PR-104A cytotoxicity (*p* < 0.01). Graph includes data from HCEC1CT, HCEC2CT, HT29, SW620, and GP2d cells with (open symbols) and without (closed symbols) SF preconditioning. Cell lines with no AKR1C3 protein (HCT116, SW480) have been removed. *: significant shift in EC_50_ upon SF preconditioning according to extra sum-of-squares F test to test if dose-response curves in control and SF pretreated cell statistically differ from each other.

## Discussion

Despite the availability of data concerning the impact of SF on gene expression, there was only limited information available concerning protein abundance alteration in human colon cells, especially for SF concentrations too low to significantly alter cell viability. In the proteomic screen conducted here, 23 proteins were differentially abundant, 18 being increased in abundance upon treatment with 2.5 μM SF. Several of these proteins, including different AKRs, were found previously to be induced by higher doses of SF (5 and 15 μM) in breast cell lines established from mammary glands with fibrocystic disease and human colon adenocarcinoma Caco-2 cells also by using proteomic approaches [14, 16]. However, in the present study, the cellular response was analyzed at a demonstrated non-toxic and potentially physiologically relevant concentration of SF exposure in human tissues (2.5 μM) [46]. At high doses, SF has cytostatic and cytotoxic activity on cultured cells [47–50]. A moderate change in AKR1C3 abundance (7.3-fold) and generally limited list of up-regulated enzymes detected in our study may be related to cell type or the low SF concentration, supporting an increase in a high proportion of reductase enzymes even at low concentration.

On the basis of the SILAC results, we explored the potential for SF preconditioning of HT29 cells to alter the cytotoxicity of the bioreductive prodrug PR-104A, and found there to be a positive interaction directly related to modulation of AKR1C3. Western blot analysis, specific enzyme activity measurements, and siRNA knock-down experiments all corroborated that enhanced sensitivity to PR-104A was related to an increase in AKR1C3 abundance levels and activity. In contrast, this sensitization was not detectable in immortalized HCEC1CT cells, consistent with the lack of induced enzyme activity detected in these cells (Fig 4A). On the basis of western blot data, there appeared to be a relative increase in protein abundance (Fig 4B). However absolute protein levels remained so low in both pre-conditioned and untreated cells that no effective sensitization was observed. The result for the HCEC1CT cell line was of interest due to its non-cancer tissue origin and molecular characteristics suggesting it as a model for healthy colon epithelial cells [38].

The possible selective enhancement of PR-104A in tumor cells motivated us to examine five additional cell lines, namely HCEC2CT (non-cancerous), and colon cancer cell lines SW480, SW620, HCT116, and GP2d to better understand how molecular factors influence the combination response. Similar to HT29, SW620 cells were sensitized towards the treatment with PR-104A by the SF-pretreatment. Furthermore, AKR1C3 protein abundance and activity was likewise induced in these cells. In contrast, protein abundance and activity was not affected by SF-pretreatment in non-cancerous HCEC2CT cells, therefore these cells were not sensitized towards the drug. In cell lines that had very low or no AKR1C3 mRNA levels [51] and did not express the protein (SW480, HCT116), AKR1C3 abundance was also not inducible with SF preconditioning. Therefore, there was no impact on PR-104A cytotoxicity. GP2d cells had low *AKR1C3* mRNA levels [51], but protein abundance and enzyme activity could be increased upon SF preconditioning, yet with a level of induction seemingly insufficient to impact the cytotoxicity of PR-104A. Thus, AKR1C3 activity appeared to be more diagnostic of PR-104A cytotoxicity (Fig 4C) than small protein abundance changes that could be detected by western blot but were insufficient to have a real effect. This observation lends additional support in the context of colon cancer cells to the suggestion that AKR1C3 activity could be used as a predictive marker for PR-104A susceptibility as put forth previously on the basis of data from patient-derived xenografts and leukemia cells. Finally, it suggests a value for continued development of strategies to monitor AKR1C3 activity, including activity-based protein profiling [30, 52, 53].

It has been speculated that additional factors, such as DNA repair, may account for inconsistent results observed previously concerning the relationship between PR-104A susceptibility and AKR1C3 activity in cell lines, however, our data suggest a weak role if any for repair differences to be significant in the colon cancer cells considering to results obtained with CBL (Table 2). PR-104A forms ICLs, proposed to be similar to those described for CBL and MMC [26–28, 54]. When we tested CBL as a model crosslinking agent and analyzed cytotoxicity in the same cell lines as PR-104A, there was no significant influence of SF preconditioning on cell viability (Table 2). However, under aerobic conditions, as employed here, PR-104A ICLs are thought to be only formed if AKR1C3 is expressed, otherwise other modes of action in cell lines lacking this enzyme have been suggested, such as monoalkylation of DNA by PR-104A itself or its oxidized half mustard [27, 28, 54]. The structures for such DNA adducts have not been rigorously characterized, nor their corresponding mode of repair known, therefore the importance of these pathways in influencing cytotoxicity in cells with low basal or induced AKR1C3 levels warrants further study.

The possibility of enhancing the cytotoxicity of bioreductive anticancer prodrugs with dietary phytochemicals has been demonstrated in a handful of previous studies of human cell lines [21, 22, 43, 55]. Four cell lines, of colon, lung or breast origin, were sensitized towards the bioreductive drug MMC by induction of NQO1 [22]. Yu *et al*. used curcumin and resveratrol to induce the drug-metabolizing enzyme PTGR1 and therefore sensitize liver HepG2 and colon SW620 cells towards the bioreductive drug hydroxymethylacylfulvene [43]. A similar sensitization towards hydroxymethylacylfulvene was obtained in HT29 cells by using D3T as enzyme inducer [55]. Additionally, there are some *in vivo* data available, for example, dietary fish oil increased NQO1 levels in human breast carcinoma tumor xenografts in mice and increased the MMC sensitivity of these tumors [56]. Human colon tumors (implanted HCT116 cells) were sensitized towards MMC by feeding mice the NQO1 inducer dimethyl fumarate [23]. To our knowledge, the effect of SF on the efficacy of bioreductive anticancer prodrugs, especially PR-104, has not been investigated previously. SF is bioavailable in the colon [4] and eating cruciferous plants seems to have a high margin of safety [57], suggesting the combination of SF with PR-104 may promote efficacy. Further studies to evaluate the robustness and selectivity of the response *in vivo* are warranted, but not trivial in a model organism because biotransformation of PR-104 has been shown to differ in humans vs. rodents, consistent with rodents not expressing a homolog of AKR1C3 [58].

In this study, preconditioning HT29 colon cancer cells with a low concentration of SF led to selective protein abundance changes within a small number of proteins. A specific cellular protein abundance and enzymatic activity increase for the reductase enzyme AKR1C3 was of particular interest as it had a direct positive effect on the cytotoxicity of the nitrogen mustard bioreductive prodrug PR-104A, but not in immortalized non-cancerous human colonic epithelial cells. In addition to AKR1C3-mediated drug activation, cellular factors differing amongst colon cell types appears to influence the efficiency of SF-promoted enzyme induction and drug sensitization, suggesting potential individual differences in responsiveness that may be detected by biomonitoring AKR1C3 expression or function, or by future identification of additional biomarkers of responsiveness. The data indicate that dietary bioactive food components such as SF, even at physiologically feasible concentrations, should be considered for their potential to alter and possibly benefit bioreductive cancer drug activity.

## Supporting Information

S1 Appendix Detailed protocol for SILAC and SF uptake experiments. (DOCX)

S1 Fig Full coumberone metabolism in all colon cell lines used in this study with (gray) and without (black) SF preconditioning. Bars correspond to mean values and error bars are standard errors. Statistical analysis was performed by an unpaired t-test with Welch’s corrections; **: *p* < 0.01, *: *p* < 0.05.
(DOCX)

S2 Fig Full dose-response curve for 48 hours SF treatment in HT29 cells. The calculated IC_50_ corresponds to 16.7 μM with a 95% confidence interval of 14.3 to 19.6. 2.5 μM correspond to an IC_10_.
(DOCX)

S3 Fig Time course of accumulation of SF in HT29 and HCEC1CT cells. For each assay, cells were exposed to 2.5 or 5 μM SF for specified times at 37°C. At the end of exposure, cells were quickly harvested, separated from medium and lysed, and the content of isothiocyanate in the lysate was measured by cyclocondensation assay (see S1 Appendix). Data is from duplicate samples except; HT29 2.5 μM SF 10 and 30 min, and HT29 5 μM 30 min for which data is from a single sample.
(DOCX)

S1 Table Full list of the quantified proteins in the SILAC experiment with their normalized H/L ratio. Column A, B and C: normalized H/L ratios for the three replicate measurements. Column D: Mean of normalized H/L ratios of all three replicate measurements. Column E: Identifiers of proteins contained in the protein group. Column F: Identifiers of proteins that have at least half of the peptides that the leading protein has. Column G: p-value upon statistical testing. Column H: false discovery rate-adjusted p-value.
(XLSX)

S2 Table Relative value for quantification of western blots showing levels of AKR1C3 protein in HT29 cells treated with either non-targeting siRNA or siAKR1C3 with or without simultaneous SF treatment (control = 0.1% DMSO). One representative western blot is shown in Fig 3D. Densitometry analysis was done using ImageJ software. Relative expression and 95% confidence interval was calculated for three independent replicates, normalized to a value of 1.0 for non-targeting DMSO sample.
(DOCX)

S3 Table Relative value for quantification of western blots showing levels of AKR1C3 protein in seven cell colon cell lines treated with or without 2.5 μM SF for 48 h (control = 0.1% DMSO). One representative western blot is shown in Fig 4B. Densitometry analysis was done using ImageJ software. Relative expression and 95% confidence interval was calculated for three independent replicates, normalized to a value of 1.0 for HT29 DMSO sample.
(DOCX)
