# Supplementary material for: Sulforaphane Preconditioning Sensitizes Human Colon Cancer Cells towards the Bioreductive Anticancer Prodrug PR-104A
Source: PLoS One. 2016 Mar 7;11(3):e0150219. doi: 10.1371/journal.pone.0150219 (PMC4780774; doi:10.1371/journal.pone.0150219)
Supplement: S1 Appendix — (DOCX) [file pone.0150219.s001.docx]

**S1 Appendix. Detailed protocol for SILAC and SF uptake experiments.**

*SILAC*

*SILAC Protein Digestion.* Equal amounts of protein extracts from SF-treated and untreated cells were mixed to obtain 75 μg total protein extract. The extract was re-suspended in 8 M urea in 50 mM ammonium bicarbonate (pH 7.8), reduced with 5 mM dithiothreitol (DTT, final concentration) for 30 min at 37 °C, and alkylated with 15 mM iodoacetamide (final concentration) at RT for 30 min in the dark. To stop the reaction, 15 mM DTT (final concentration) was added. After 10 min, samples were diluted with 50 mM ammonium bicarbonate (pH 7.8) to a final concentration of 0.6 M urea, and 3% (v/v) methanol was added. Proteins were digested with sequencing grade modified trypsin (1:50 trypsin to protein ratio, Promega) at 37 °C for 6 h. Digestion was stopped by adding 0.5% (v/v) trifluoroacetic acid (TFA, final concentration).

*Fractionation with High-Performance Liquid Chromatography.* Peptides from protein extract digests were desalted with Sep-Pak C18 (Waters), lyophilized to dryness, dissolved in 1 mL 25 mM K_2_HPO_4_ (pH 11.0) with 5% (v/v) acetonitrile (ACN), and injected on a YMC-Triart C18 column (150 x 4.6 mm, 5 μm) connected to an Agilent HPLC 1100 system. Mobile phases A and B were 5% (v/v) ACN in 25 mM K_2_HPO_4_ and 50% (v/v) ACN in 25 mM K_2_HPO_4_ pH 11. Sample loading was performed at 1 mL/min with 2% B for 10 min followed by a linear gradient from 2 to 10% B over 5 min, 10 to 50% B over 25 min, and a 5 min hold at 98% B prior to re-equilibration at 2% B. Elution was monitored by absorbance at 214 nm and peptides were fractionated into 12 samples, lyophilized and re-suspended in 3% (v/v) ACN acidified with 0.1% TFA. Pooled fractions were desalted with a C18 ZipTip (Millipore), lyophilized and re-suspended in 3% (v/v) ACN with 0.1% formic acid.

*Liquid Chromatography-Tandem Mass Spectrometry (LC-MS/MS)*. One μg of each HPLC fraction was analyzed with LC-ESI-MS/MS. Peptides were separated on a 75 μm fused silica emitter packed with 8 cm Magic C18 AQ 3 μm (Michrom Bioresources) nanoLC system (Eksigent). The following LC gradient was applied: 0 min: 0.8% mobile phase B, 3 min: 4.8% B, 52 min: 32.8% B, 55 min: 48% B, 60 min: 96.8% B, 71 min: 96.8% B. Mobile phases A and B were 1% ACN and 100% ACN, 0.2% FA. Ionized tryptic peptides were detected on an LTQ Orbitrap Velos mass spectrometer (Thermo Fisher Scientific) with one full scan MS (m/z 300-1700) at a resolution of 60’000 at m/z 400 of which the 15 most intense precursor ions were fragmented by collision-induced dissociation in the ion trap. Charge state screening was enabled, and singly charged precursor ions and signals with undefined charge states were excluded. The dynamic exclusion list was restricted to a maximum of 500 entries with maximum exclusion duration of 45 s and a relative mass window of ± 5 ppm.

The raw MS data were analyzed with MaxQuant 1.5.0.0 ([1](#_ENREF_1), [2](#_ENREF_2)). Full tryptic specificity was required with up to two missed cleavages. Carbamidomethylation of cysteine was set as a fixed modification, with oxidation of methionine and protein N-terminal acetylation as variable modifications. Precursor and fragment ion mass tolerances were 6 ppm and 0.5 Da. We used the UniProt KB human protein database containing 20,207 protein sequences (downloaded June 2014) and common protein contaminants. The “match between runs” option was enabled for 2 min as well as the “requantify” option. The FDR was 1% on the peptide and 5% on the protein level. Proteins identified by site, as decoys or contaminants were filtered out. For quantification, at least one unique peptide per protein and a minimum of one heavy-to-light (H/L) ratio count was required in each replicate. The normalized H/L ratios of the three replicates were log_2_ transformed and significance was assessed in a two-sided t-test using Perseus 1.5.0.15 ([3](#_ENREF_3)). P-values were adjusted for multiple testing using the Benjamini-Hochberg method ([4](#_ENREF_4)). An adjusted p-value of less than 0.1 and a fold-change less than 0.67 or more than 1.5 were required for reporting down- or up-regulation.

*SF Uptake*

*Synthesis of 1,3-Benzodithiole-2-thione.* Compound was synthesized according to ([5](#_ENREF_5)) with some modifications. Equal volumes of 200 mM propyl isothiocyanate, 1 M 1,2-benzenedithiol (both dissolved in acetonitrile) and 0.1 M potassium phosphate buffer (pH 7) were mixed and stirred for 18 h. After centrifugation (5 min; 3000 rcf), the supernatant was removed and the precipitate dried under vacuum. Structure was confirmed by mass spectrometry and ^1^H-NMR, which matched the published literature values ([5](#_ENREF_5), [6](#_ENREF_6)).

*Preparation of Cell Lysate for Determination of SF Uptake.* 6 x 10^5^ cells were plated in a 10 cm dish with 10 mL medium. After 48 h, cell layers were exposed to SF for indicated time periods. At the end of the exposure, cells were washed and trypsinized (1 mL). Cells were resuspended in 10 mL medium and 10 mL of this suspension were quickly transferred to a 15 mL tube containing cold 2 mL of equal volumes of dibutyl phthalate and diisononyl phthalate and centrifuged at 1800 rcf at 4 °C for 2 min to separate the cells from the medium. The medium and oil were removed by aspiration and the entire pellet was then resuspended in 500 μL H_2_O, frozen in liquid nitrogen and stored at -80 °C until analysis. The remaining 1 mL of the cell suspension was used to determine the packed cell volume (PCV) using PCV Packed Cell Volume Tubes (TPP Techno Plastic Products AG, Trasadingen, Switzerland). Cellular SF concentrations in the different cell lines were measured by the HPLC-coupled cyclocondensation assay (reaction with 1,2-benzenedithiol), as previously described ([5](#_ENREF_5), [7](#_ENREF_7), [8](#_ENREF_8)).

*Cyclocondensation Reaction Procedure.* The cyclocondensation reactions were carried out in glass vials. 2 mL reaction mixture consisted of 500 μL 100 mM potassium phosphate buffer (pH 8.5), 1 mL 20 mM benzene-1,2-dithiol in 100% acetonitrile, and 500 μL of the sample to be analyzed. The solution was heated for 2 h at 65 °C. After cooling to room temperature, the mixture was centrifuged at low speed to remove insoluble materials, and a 1.5-mL portion of the supernatant fraction was mixed with 1.5 mL of water. This solution was loaded on a small Sep-Pak C_18_ Cartridge (Waters). The cartridge was then washed twice with 1 mL 25% acetonitrile and subsequently eluted with 300 μL acetonitrile. 100 μL of the eluent were injected into the HPLC system.

*HPLC.* An Agilent Technologies 1200 HPLC solvent delivery system was used to quantitate 1,3-benzodithiole-2-thione, the cyclocondensation product of 1,2-benzenedithiol with isothiocyanates. An analytical C_18_ reverse-phase column (Eclipse XDB-C18, 5 μm, 4.6 x 150 mm; Agilent Technologies) was used and was operated isocratically with 85% acetonitrile/15% water containing 0.1% TFA, at a rate of 1 mL/min. The eluents were monitored at 365 nm by the photodiode array detector and the area of the 1,3-benzodithiole-2-thione peak (eluted at 3.2 min) was integrated. A 17-min time interval was assigned for column washing and equilibration between successive sample injections. For quantification of the peak, a calibration curve with synthetic 1,3-Benzodithiole-2-thione has been performed. The packed cell volume (PCV) measure before cell lysis was used to determine cellular SF uptake.

**References**

1. Cox J, Mann M. MaxQuant enables high peptide identification rates, individualized p.p.b.-range mass accuracies and proteome-wide protein quantification. Nat Biotechnol 2008;26:1367-72.

2. Cox J, Neuhauser N, Michalski A, Scheltema RA, Olsen JV, Mann M. Andromeda: A Peptide Search Engine Integrated into the MaxQuant Environment. J Proteome Res 2011;10:1794-805.

3. Cox J, Mann M. 1D and 2D annotation enrichment: a statistical method integrating quantitative proteomics with complementary high-throughput data. BMC Bioinformatics 2012;13:S12.

4. Benjamini Y, Hochberg Y. Controlling the False Discovery Rate: a Practical and Powerful Approach to Multiple Testing. J R Statist Soc B 1995:289-300.

5. Zhang Y, Cho C-G, Posner GH, Talalay P. Spectroscopic Quantitation of Organic Isothiocyanates by Cyclocondensation with Vicinal Dithiols. Anal Biochem 1992;205:100-7.

6. Smith K, Lindsay CM, Pritchard GJ. Directed Lithiation of Arenethiols. J Am Chem Soc 1989;111:665-9.

7. Zhang Y, Wade KL, Prestera T, Talalay P. Quantitative Determination of Isothiocyanates, Dithiocarbamates, Carbon Disulfide, and Related Thiocarbonyl Compounds by Cyclocondensation with 1,2-Benzenedithiol. Anal Biochem 1996;239:160-7.

8. Ye L, Dinkova-Kostova AT, Wade KL, Zhang Y, Shapiro TA, Talalay P. Quantitative determination of dithiocarbamates in human plasma, serum, erythrocytes and urine: pharmacokinetics of broccoli sprout isothiocyanates in humans. Clin Chim Acta 2002;316:43-53.
