# Supplementary material for: Sulforaphane Preconditioning Sensitizes Human Colon Cancer Cells towards the Bioreductive Anticancer Prodrug PR-104A
Source: PLoS One. 2016 Mar 7;11(3):e0150219. doi: 10.1371/journal.pone.0150219 (PMC4780774; doi:10.1371/journal.pone.0150219)
Supplement: S1 Fig — Bars correspond to mean values and error bars are standard errors. Statistical analysis was performed by an unpaired t-test with Welch’s corrections; **: p < 0.01, *: p < 0.05. (DOCX) [file pone.0150219.s002.docx]

**

**

Figure S1. Full coumberone metabolism in all colon cell lines used in this study with (gray) and without (black) SF preconditioning. Bars correspond to mean values and error bars are standard errors. Statistical analysis was performed by an unpaired t-test with Welch’s corrections; **: *p* < 0.01, *: *p* < 0.05.
