# Supplementary material for: Sulforaphane Preconditioning Sensitizes Human Colon Cancer Cells towards the Bioreductive Anticancer Prodrug PR-104A
Source: PLoS One. 2016 Mar 7;11(3):e0150219. doi: 10.1371/journal.pone.0150219 (PMC4780774; doi:10.1371/journal.pone.0150219)
Supplement: S2 Fig — The calculated IC50 corresponds to 16.7 μM with a 95% confidence interval of 14.3 to 19.6. 2.5 μM correspond to an IC10. (DOCX) [file pone.0150219.s003.docx]

**

**

Figure S2. Full dose-response curve for 48 hours SF treatment in HT29 cells. The calculated IC_50_ corresponds to 16.7 μM with a 95% confidence interval of 14.3 to 19.6. 2.5 μM correspond to an IC_10_.
