# Supplementary material for: Sulforaphane Preconditioning Sensitizes Human Colon Cancer Cells towards the Bioreductive Anticancer Prodrug PR-104A
Source: PLoS One. 2016 Mar 7;11(3):e0150219. doi: 10.1371/journal.pone.0150219 (PMC4780774; doi:10.1371/journal.pone.0150219)
Supplement: S3 Fig — For each assay, cells were exposed to 2.5 or 5 μM SF for specified times at 37°C. At the end of exposure, cells were quickly harvested, separated from medium and lysed, and the content of isothiocyanate in the lysate was measured by cyclocondensation assay (see S1 Appendix). Data is from duplicate samples except; HT29 2.5 μM SF 10 and 30 min, and HT29 5 μM 30 min for which data is from a single sample. (DOCX) [file pone.0150219.s004.docx]

**
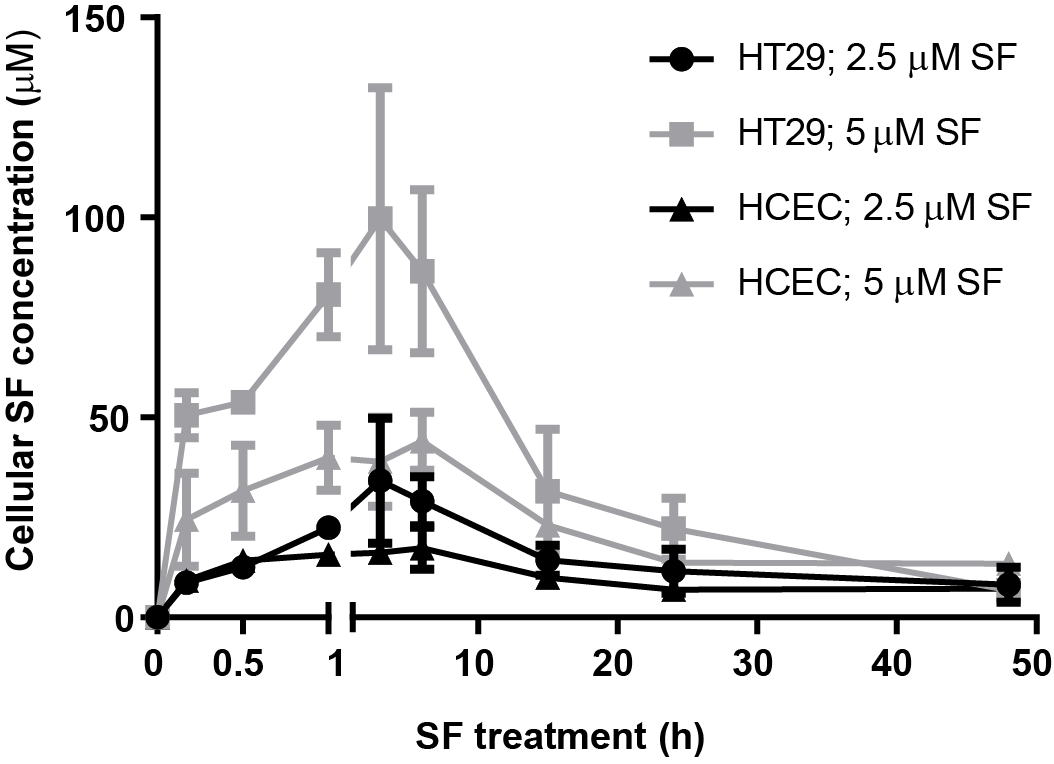
**

Figure S3. Time course of accumulation of SF in HT29 and HCEC1CT cells. For each assay, cells were exposed to 2.5 or 5 μM SF for specified times at 37 °C. At the end of exposure, cells were quickly harvested, separated from medium and lysed, and the content of isothiocyanate in the lysate was measured by the cyclocondensation assay (see Supplementary Information Materials and Methods). Samples have been prepared in duplicate, except for HT29; 2.5 μM SF 10 and 30 min and for HT29; 5 μM 30 min only one replicate has been measured.
