# Supplementary material for: Sulforaphane Preconditioning Sensitizes Human Colon Cancer Cells towards the Bioreductive Anticancer Prodrug PR-104A
Source: PLoS One. 2016 Mar 7;11(3):e0150219. doi: 10.1371/journal.pone.0150219 (PMC4780774; doi:10.1371/journal.pone.0150219)
Supplement: S2 Table — One representative western blot is shown in Fig 3D. Densitometry analysis was done using ImageJ software. Relative expression and 95% confidence interval was calculated for three independent replicates, normalized to a value of 1.0 for non-targeting DMSO sample. (DOCX) [file pone.0150219.s006.docx]

Table S2. Relative value for quantification of western blots showing levels of AKR1C3 protein in HT29 cells treated with either non-targeting siRNA or siAKR1C3 with or without simultaneous SF treatment (control = 0.1% DMSO). One representative western blot is shown in Figure 3d. Densitometry analysis was done using ImageJ software. Relative expression and 95% confidence interval was calculated for three independent replicates, normalized to a value of 1.0 for non-targeting DMSO sample.

| siRNA target | Treatment (48h) | AKR1C3 expression  (relative to expression in non-targeting DMSO) | 95% CI |
| --- | --- | --- | --- |
| non-targeting | 0.1% DMSO | 1.00 |  |
| non-targeting | 2.5 μM SF | 4.90 | ­-1.09—10.89 |
| AKR1C3 | 0.1% DMSO | 0.13 | ­-0.17—0.43 |
| AKR1C3 | 2.5 μM SF | 0.62 | 0.03—1.21 |
