# Supplementary material for: Sulforaphane Preconditioning Sensitizes Human Colon Cancer Cells towards the Bioreductive Anticancer Prodrug PR-104A
Source: PLoS One. 2016 Mar 7;11(3):e0150219. doi: 10.1371/journal.pone.0150219 (PMC4780774; doi:10.1371/journal.pone.0150219)
Supplement: S3 Table — One representative western blot is shown in Fig 4B. Densitometry analysis was done using ImageJ software. Relative expression and 95% confidence interval was calculated for three independent replicates, normalized to a value of 1.0 for HT29 DMSO sample. (DOCX) [file pone.0150219.s007.docx]

Table S3. Relative value for quantification of western blots showing levels of AKR1C3 protein in seven cell colon cell lines treated with or without 2.5 μM SF for 48 h (control = 0.1% DMSO). One representative western blot is shown in Figure 4b. Densitometry analysis was done using ImageJ software. Relative expression and 95% confidence interval was calculated for three independent replicates, normalized to a value of 1.0 for HT29 DMSO sample.

| Cell line | Treatment (48h) | AKR1C3 expression  (relative to HT29/DMSO) | 95% CI |
| --- | --- | --- | --- |
| HCEC1CT | 0.1% DMSO | 0.04 | ­-0.02—0.09 |
| HCEC1CT | 2.5 μM SF | 0.19 | ­-0.02—0.40 |
| HCEC2CT | 0.1% DMSO | 2.31 | 0.85—3.78 |
| HCEC2CT | 2.5 μM SF | 4.43 | 0.57—8.30 |
| HT29 | 0.1% DMSO | 1.00 |  |
| HT29 | 2.5 μM SF | 6.55 | 2.99—10.12 |
| SW620 | 0.1% DMSO | 1.19 | ­-0.49—2.89 |
| SW620 | 2.5 μM SF | 3.11 | ­-0.89—7.11 |
| SW480 | 0.1% DMSO | 0.00 | 0 |
| SW480 | 2.5 μM SF | 0.00 | 0 |
| HCT116 | 0.1% DMSO | 0.00 | 0 |
| HCT116 | 2.5 μM SF | 0.00 | 0 |
| GP2d | 0.1% DMSO | 0.27 | ­-3.16—3.70 |
| GP2d | 2.5 μM SF | 0.45 | ­-4.76—5.66 |
